# APPLIED METHODS FOR HEALTH EQUITY AND END-OF-LIFE OUTCOMES RESEARCH USING MEDICARE DATA

**DOI:** 10.1093/geroni/igad104.1462

**Published:** 2023-12-21

**Authors:** Olga Jarrín, Hyosin Kim

## Abstract

This symposium brings together four related and complementary projects on the development of new data sources and methods to analyze the new data to study health equity and end-of-life outcomes research. The first abstract presents the development of the Care Setting Trajectory Database, which integrates data from various CMS data sources for Medicare beneficiaries to create a longitudinal care experience for older adults across healthcare settings. The second abstract describes the creation of a new race/ethnicity variable to augment the Medicare administrative data for minority populations using harmonized, self-reported race/ethnicity data. The third abstract describes the development of harmonized datasets to study geriatric syndromes’ prevalence, cost, and health outcomes over time. The fourth abstract identifies and predicts place of care trajectories during the last three years of life among Medicare beneficiaries, identifying dual trajectories of inpatient/institutional care and skilled home healthcare/home hospice. Finally, the fifth abstract includes an application of the care setting trajectory database and our new, harmonized, self-reported race/ethnicity variable to evaluate the impact of home health care on later use of hospice and place of death in a large cohort of Medicare decedents. This symposium introduces innovative approaches for harmonizing and merging CMS data sources for Medicare beneficiaries. The methodological advances described in this symposium aim to support health services researchers and policymakers to generate and evaluate research improving care coordination, continuity, and quality of life for older adults with serious illnesses.

